# Prediction of cholesterol ratios within a Korean population

**DOI:** 10.1098/rsos.171204

**Published:** 2018-01-17

**Authors:** Jin Sol Lee, Hyun Sub Cheong, Hyoung Doo Shin

**Affiliations:** 1 Department of Life Science, Sogang University, Baekbumro 35, Mapo-gu, Seoul 04107, Republic of Korea; 2 Research Institute for Basic Science, Sogang University, Mapo-gu, Seoul, 121-742, Republic of Korea; 3 Department of Genetic Epidemiology, SNP Genetics, Inc., Taihard building 1007, Sogang University, Baekbumro 35, Mapo-gu, Seoul, Republic of Korea

**Keywords:** prediction, total cholesterol, triglyceride, HDL

## Abstract

Cholesterol ratios (total cholesterol (TC)/high-density lipoprotein cholesterol (HDL-c) and triglyceride (TG)/HDL-c) have been suggested as better indicators to predict various clinical features such as insulin resistance and heart disease. Therefore, we aimed to build a single nucleotide polymorphism (SNP) set to predict constitutional lipid metabolism. The genotype data of 7795 samples were obtained from the Korea Association Resource. Among the total of 7795 samples, 7016 subjects were used to perform 10-fold cross-validation. We selected the SNPs that showed significance constantly throughout all 10 cross-validation sets; another 779 samples were used as the final validation set. After performing the 10-fold cross-validation, the six SNPs (*rs4420638* (*APOC1*), *rs12421652* (*BUD13*)*, rs17411126* (*LPL*)*, rs6589566* (*ZPR1*)*, rs16940212* (*LOC101928635*) and *rs10852765* (*ABCA8*)) were finally selected for predicting cholesterol ratios. The weighted genetic risk scores (wGRS) were calculated based on the regression slopes of the six selected SNPs. Our results showed upward trends of wGRS for both the TC/HDL-c and TG/HDL-c ratios within the 10-fold cross-validation. Similarly, the wGRS of the six SNPs also showed upward trends in analyses using the SNP selection set and final validation set. The selected six SNPs can be used to explain both the TC/HDL-c and TG/HDL-c ratios. Our results may be useful for the prospective predictions of cholesterol-related diseases.

## 1. Introduction

Blood cholesterol and lipids are well-known heritable risk factors of cardiovascular diseases, including heart attacks and stroke [1,2]. Therefore, numerous large-scale genetic studies have been conducted to identify cholesterol and lipid-associated markers. One result of these efforts is that many significantly lipid-related markers have been revealed. For example, one recent genome-wide association study (GWAS) found new lipid-associated markers such as *CD163*-*APOBEC1*, *NCOA2*, *NID2*-*PTGDR* and *WDR11*-*FGFR2* [3].

It was suggested that blood cholesterol ratios that use total cholesterol (TC), triglyceride (TG), and high-density lipoprotein cholesterol (HDL-c) are more effective indicators for the prediction of various cardiovascular diseases compared to the traditional lipid level [4]. For example, TC and serum lipoprotein ratios were associated with blood pressure [5]. Other previous studies have also reported that the TC/HDL-c ratio was a more effective marker for coronary heart disease risk [6,7]. In addition, the TG to HDL-c ratio was an important marker for insulin resistance, which was related to type 2 diabetes mellitus, particularly in a rural Korean population [8]. Several other previous studies have supported the implications of TG and HDL-C in insulin resistance [9–11]. Moreover, TG/HDL-c ratios were reported to be possible indicators of low-density lipoprotein cholesterol particle size in patients with type 2 diabetes and normal HDL-c levels [12].

Considering the effect of cholesterol ratios on clinical features, predicting cholesterol ratios could help increase the quality of life. However, previous studies have focused on the finding of markers for traditional lipid levels. Indeed, there was only one GWAS for cholesterol ratios with significant markers in the Korean population [13].

We investigated a single nucleotide polymorphism (SNP) set in the present study to predict cholesterol ratios with the weighted genetic risk score (wGRS) method using the genotype data from the Korea Association Resource (KARE). The wGRS method is a simple widely used method for building a set of SNPs for prediction. Several previous studies have already shown the usefulness of wGRS as a prediction model for various diseases [14–16]. Moreover, we only used previously reported significant SNPs in GWAS to increase our study's validity. A further 10-fold cross-validation process was also performed to select constantly significant SNPs in all analysis sets.

## 2. Method

### 2.1. Study subjects

The present study used the genotype data from the KARE project. This study was approved by the Public Institutional Bioethics Committee as designated by the Ministry of Health and Welfare (P01-201502-31-002). Regarding the quality of the genotype data, we deleted samples and SNPs that showed a call rate lower than 98%, and SNPs with a minor allele frequency (MAF) of less than 0.05 were also excluded in further analyses. Finally, 7795 samples in total (3675 males and 4120 females) were used for the statistical analyses. The 7795 samples were divided into one set of 7016 samples (3308 males and 3708 females) as a part of the SNP selection set for 10-fold cross-validation and the remaining 779 samples (367 males and 412 females) were used as the final validation set. The statistical powers of this study were obtained using G*Power Version 3.1 software (Universität Kiel, Germany) [17]. The software calculated both the test set (*n* = 702) and the final validation set (*n* = 779) as at over 95%. Details about the number of samples are as shown in table 1.

**Table 1.** Clinical characteristics of each analysis group. Average clinical traits of analysis groups including total, SNP selection for 10-fold cross-validation and final validation set; BMI, body mass index; HDL-c, high-density lipoprotein cholesterol.

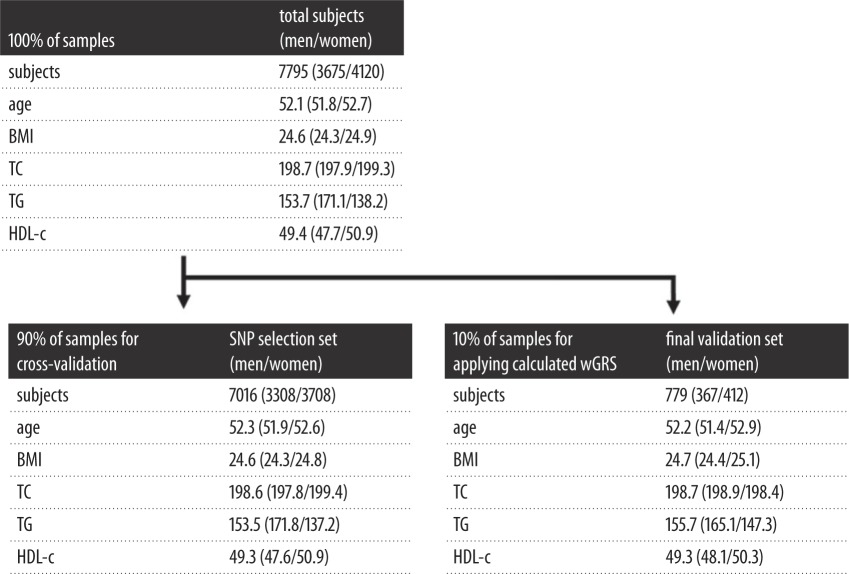

### 2.2. SNP pruning for statistical analyses

First, we collected 351 significant SNPs that had been reported in previous cholesterol-related GWAS with a secondary replication study to identify reliable SNPs for cholesterol ratio prediction [18–22]. Then, we obtained the genotype data of the collected GWAS catalogue markers including other markers in nearby regions (±100 kb from the GWAS markers) from the KARE data (7103 SNPs). The linkage disequilibrium (LD) coefficients (*r*^2^ > 0.2) of all pairs of SNPs were calculated using the Haploview software to prevent the issue in the wGRS method that is caused by high LD [23]. Among the 7103 SNPs, a set of 691 markers were remained after LD calculation. Then, we excluded SNPs which were not linked (*r*^2^ < 0.98) to previous reported GWAS catalogue SNPs. Finally, we obtained 134 SNPs for further analyses.

### 2.3. SNP selection for cholesterol prediction

From the SNP selection set (7016 samples), 10-fold cross-validation was conducted on the genotype data (the training set of 6314 subjects and the test set of 702 subjects) to identify the SNPs that could be used for cholesterol prediction. Log-transformed TG values were used for statistical analyses. The *p*-values of the SNPs were obtained via regression analyses using the training set (*n* = 6314) to identify the most significant SNPs. Regression analysis was conducted using the GoldenHelix SVS8 software (Bozeman, MT, USA). Three clinical values (age, sex and body mass index, BMI) were used as covariates. The most significant SNPs in the same LD were selected for each training set. To improve the validity of the present study, we used only SNPs which showed *p*-values lower than 0.01 in statistical analyses. The wGRS was calculated as the sum of the number of cholesterol ratio-increasing alleles multiplied by the regression slope across all variants in each set, as previously described ($\sum_{i=1}^{n}{\text{number of risk allele in SNP}}_{i}\times {\text{weight}}_{i}$; *n* = number of SNP, weight: regression slope value of SNP*_i_*) [24]. Then, we divided the cholesterol ratios of each set into quartiles and calculated the average wGRS. After 10-fold cross-validation, we selected six SNPs that overlapped across all training sets (electronic supplementary material, table S1). We applied wGRS in the quartile of the validation set that had the same cholesterol ratios as the SNP selection set to observe wGRS variation.

## 3. Results

The average age, BMI, TC and HDL-c were higher in female subjects than male subjects in overall subjects (age, 51.8 and 52.7; BMI, 24.3 and 24.9; TC 197.9 and 199.3; HDL-c, 47.7 and 50.9 in men and women, respectively). Similar results were observed in the SNP selection set and the final validation set. By contrast, TG was higher in male than in female subjects (171.1 for men and 138.2 for women overall). Detailed information about the clinical characteristics was shown in table 1.

**Figure 1.**
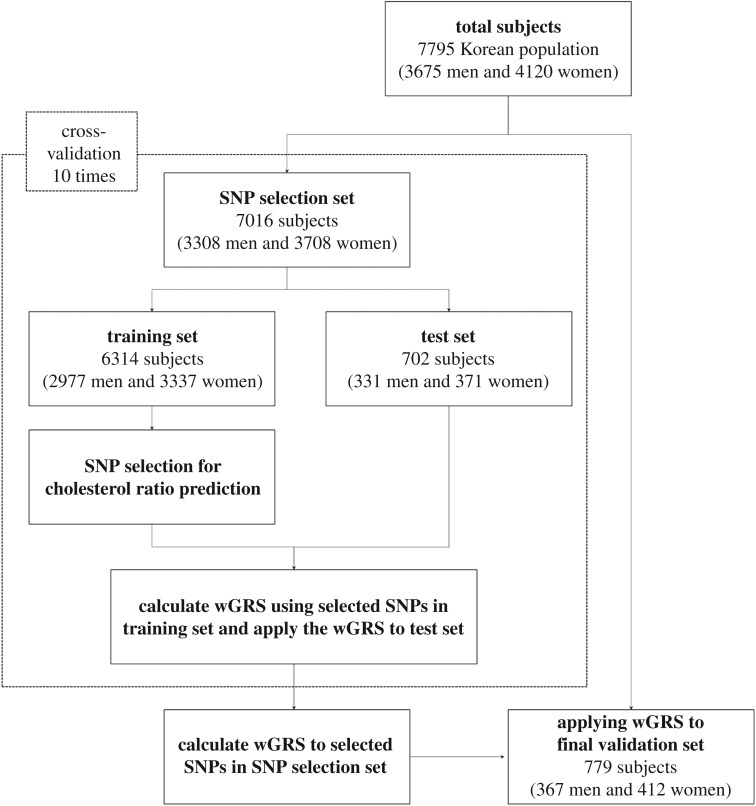
Analysis flow chart for prediction of cholesterol ratios.

The analysis process for the cholesterol ratio prediction was summarized in figure 1. Among all GWAS catalogue and nearby SNPs (around 100 kb), the twelve SNPs (*rs4420638*, *rs6589566*, *rs12421652*, *rs17411126*, *rs16940212*, *rs10852765*, *rs12229654*, *rs1250252*, *rs12686004*, *rs164212*, *rs2297194* and *rs496311*) were reached at our *p*-value threshold (*p* < 0.01) for both the TC/HDL-c and TG/HDL-c ratios (table 2). We performed 10-fold cross-validation by randomly dividing 7016 samples of the SNP selection set into 6314 samples as a training set and 702 samples as a test set. The 10-fold cross-validation process identified that only six SNPs (*rs4420638* (*APOC1*), *rs12421652* (*BUD13*)*, rs17411126* (*LPL*)*, rs6589566* (*ZPR1*)*, rs16940212* (*LOC101928635*) and *rs10852765* (*ABCA8*)) constantly showed significance in all 10 training sets (the highest *p*-value was 0.01 for *rs10852765* in sets 8 and 9) (table 2). Detailed information of the selected six SNPs is listed in table 3 with their location, allele information and genotype data with their average cholesterol ratios.

**Table 2.** *p*-Values of significant 12 markers among GWAS catalogue and nearby SNPs with genotype data from Korea Association Resource. The SNPs were selected based on *p*-values of both TC/HDL and TG/HDL. The SNPs which showed *p*-values under 0.01 were bold-faced and used for further analyses. TC, total cholesterol; TG, triglyceride; HDL-c, high-density lipoprotein cholesterol.

			training sets for 10-fold cross-validation (*n* = 6314)									
markers	lipid ratio	SNP selection set (*n* = 7016)	set 1	set 2	set 3	set 4	set 5	set 6	set 7	set 8	set 9	set 10
*rs4420638*	TC/HDL-c	**4.49 × 10^−14^**	**6.64 × 10^−13^**	**6.87 × 10^−13^**	**1.81 × 10^−11^**	**6.87 × 10^−13^**	**2.70 × 10^−13^**	**2.12 × 10^−12^**	**1.09 × 10^−13^**	**6.41 × 10^−13^**	**8.74 × 10^−13^**	**5.85 × 10^−13^**
	TG/HDL-c	**3.75 × 10^−11^**	**1.65 × 10^−10^**	**9.19 × 10^−10^**	**1.54 × 10^−9^**	**9.19 × 10^−10^**	**1.21 × 10^−10^**	**2.21 × 10^−9^**	**1.16 × 10^−10^**	**5.33 × 10^−11^**	**2.49 × 10^−10^**	**1.57 × 10^−10^**
*rs6589566*	TC/HDL-c	**2.99 × 10^−8^**	**2.05 × 10^−7^**	**6.07 × 10^−8^**	**2.30 × 10^−7^**	**6.07 × 10^−8^**	**6.83 × 10^−8^**	**5.93 × 10^−8^**	**4.20 × 10^−7^**	**4.98 × 10^−7^**	**2.00 × 10^−7^**	**8.42 × 10^−8^**
	TG/HDL-c	**2.73 × 10^−21^**	**4.40 × 10^−18^**	**4.77 × 10^−19^**	**2.46 × 10^−19^**	**4.77 × 10^−19^**	**1.71 × 10^−19^**	**2.30 × 10^−19^**	**3.86 × 10^−18^**	**1.47 × 10^−17^**	**1.99 × 10^−18^**	**2.39 × 10^−18^**
*rs12421652*	TC/HDL-c	**3.42 × 10^−8^**	**6.92 × 10^−8^**	**3.25 × 10^−8^**	**7.19 × 10^−8^**	**3.25 × 10^−8^**	**8.21 × 10^−8^**	**6.71 × 10^−8^**	**1.66 × 10^−7^**	**2.49 × 10^−7^**	**2.79 × 10^−7^**	**7.23 × 10^−8^**
	TG/HDL-c	**5.17 × 10^−15^**	**2.09 × 10^−13^**	**2.01 × 10^−14^**	**2.95 × 10^−15^**	**2.01 × 10^−14^**	**7.55 × 10^−15^**	**3.84 × 10^−14^**	**6.34 × 10^−14^**	**4.98 × 10^−12^**	**2.15 × 10^−13^**	**7.50 × 10^−13^**
*rs17411126*	TC/HDL-c	**1.76 × 10^−6^**	**1.02 × 10^−6^**	**2.43 × 10^−6^**	**1.99 × 10^−6^**	**2.43 × 10^−6^**	**4.76 × 10^−6^**	**7.45 × 10^−6^**	**1.04 × 10^−5^**	**2.94 × 10^−7^**	**6.67 × 10^−6^**	**5.16 × 10^−6^**
	TG/HDL-c	**9.70 × 10^−15^**	**3.48 × 10^−14^**	**1.98 × 10^−12^**	**1.75 × 10^−14^**	**1.98 × 10^−12^**	**3.59 × 10^−13^**	**5.87 × 10^−13^**	**8.01 × 10^−13^**	**4.74 × 10^−15^**	**2.63 × 10^−13^**	**9.89 × 10^−14^**
*rs16940212*	TC/HDL-c	**0.0002**	**0**.**001**	**0**.**002**	**0.0002**	**0**.**002**	**0**.**0006**	**0**.**0002**	**0**.**001**	**0**.**0007**	**0**.**0003**	**0**.**0002**
	TG/HDL-c	**0.0002**	**0**.**0002**	**0**.**001**	**0.0002**	**0**.**001**	**0**.**00004**	**0**.**00003**	**0**.**0005**	**0**.**0006**	**0**.**0002**	**0**.**0001**
*rs10852765*	TC/HDL-c	**0.01**	**0**.**008**	**0**.**005**	**0.006**	**0**.**005**	**0**.**007**	**0**.**008**	**0**.**007**	**0**.**01**	**0**.**01**	**0**.**006**
	TG/HDL-c	**0.006**	**0**.**006**	**0**.**005**	**0.007**	**0**.**005**	**0**.**003**	**0**.**007**	**0**.**008**	**0**.**009**	**0**.**005**	**0**.**004**
*rs12229654*	TC/HDL-c	**2.71 × 10^−5^**	**0**.**0002**	**0**.**0003**	**9.50 × 10^−5^**	**0**.**0003**	**0**.**0001**	**0**.**0007**	**0**.**0005**	**0**.**0002**	**0**.**0001**	**0**.**0002**
	TG/HDL-c	**0.01**	0.02	**0**.**01**	0.02	**0**.**01**	0.02	0.03	0.03	0.03	0.02	0.03
*rs1250252*	TC/HDL-c	**0.0006**	**0**.**001**	**0**.**001**	**0.001**	**0**.**001**	**0**.**003**	**0**.**001**	**0**.**002**	**0**.**0004**	**0**.**0003**	**0**.**0004**
	TG/HDL-c	**0.01**	**0**.**009**	**0**.**01**	0.02	**0**.**01**	0.05	**0**.**01**	0.04	**0**.**003**	**0**.**003**	**0**.**002**
*rs12686004*	TC/HDL-c	**0.006**	**0**.**008**	**0**.**005**	0.02	**0**.**005**	0.02	**0**.**009**	0.04	**0**.**008**	**0**.**006**	**0**.**003**
	TG/HDL-c	**1.23 × 10^−6^**	**5.90 × 10^−6^**	**5.63 × 10^−6^**	**2.00 × 10^−5^**	**5.63 × 10^−6^**	**1.68 × 10^−5^**	**5.69 × 10^−6^**	**2.48 × 10^−5^**	**2.03 × 10^−6^**	**1.79 × 10^−6^**	**6.51 × 10^−7^**
*rs164212*	TC/HDL-c	**0.009**	**0**.**01**	**0**.**009**	**0.006**	**0**.**009**	**0**.**01**	**0**.**008**	**0**.**01**	**0**.**008**	**0**.**01**	**0**.**006**
	TG/HDL-c	**0.01**	**0**.**01**	**0**.**004**	**0.01**	**0**.**004**	**0**.**006**	**0**.**005**	**0**.**004**	0.05	0.03	0.02
*rs2297194*	TC/HDL-c	**0.01**	0.04	0.03	**0.009**	0.03	0.02	**0**.**006**	**0**.**01**	0.07	0.06	0.02
	TG/HDL-c	**2.34 × 10^−5^**	**5.01 × 10^−5^**	**4.25 × 10^−5^**	**3.30 × 10^−6^**	**4.25 × 10^−5^**	**3.81 × 10^−5^**	**1.77 × 10^−5^**	**3.36 × 10^−5^**	**0**.**0002**	**0**.**0004**	**5.76 × 10^−5^**
*rs496311*	TC/HDL-c	**0.01**	0.03	0.08	0.03	0.08	0.03	0.03	0.02	0.03	0.03	0.03
	TG/HDL-c	**0.003**	**0**.**004**	**0**.**01**	**0.006**	**0**.**01**	**0**.**007**	**0**.**009**	**0**.**004**	**0**.**004**	**0**.**005**	**0**.**008**

**Table 3.** Information of used markers for cholesterol ratio prediction. Gene name, location and position of the SNPs were listed based on NCBI database. C/C, C/R and R/R represent the homozygote of the major allele and the heterozygote and homozygote of the minor allele, respectively. LD information was obtained from 1000 Genomes project data (http://www.internationalgenome.org/). GWAS, genome-wide association study; MAF, minor allele frequency.

			allele information				genotype count with average cholesterol ratios				
markers	gene	location	minor	major	MAF		C/C	C/R	R/R	linked GWAS catalogue SNP	reference (PMID)
*rs4420638*	*APOC1*	19:44919689	G	A	0.111	genotype count	6181	1502	112	reported	Willer *et al*. [18]
						TC/HDL-c	4.163	4.402	4.485		(24097068)
						triglyceride/HDL-c	0.0451	0.0478	0.0488		
*rs6589566*	*ZPR1*	11:116781707	G	A	0.216	genotype count	4783	2649	363	reported	Kim *et al*. [13]
						TC/HDL-c	4.156	4.274	4.527		(28046027)
						triglyceride/HDL-c	0.0446	0.0471	0.0504		
*rs12421652*	*BUD13*	11:116755159	T	G	0.203	genotype count	4959	2515	321	*rs11216126*	Kim *et al*. [19]
						TC/HDL-c	4.282	4.107	4.003	(*r*^2^ = 1.00)	(21909109)
						triglyceride/HDL-c	0.0467	0.0442	0.0417		
*rs17411126*	near *LPL*	8:19997761	C	T	0.207	genotype count	4896	2564	335	*rs326*	Coram *et al*. [20]
						TC/HDL-c	4.262	4.151	3.986	(*r*^2^ = 1.00)	(23726366)
						triglyceride/HDL-c	0.0466	0.0443	0.0422		
*rs16940212*	*LOC101928635*	15:58401821	T	G	0.340	genotype count	3394	3497	904	reported	Kim *et al*. [19]
						TC/HDL-c	4.274	4.196	4.056		(21909109)
						triglyceride/HDL-c	0.0465	0.0456	0.0433		
*rs10852765*	*ABCA8*	17:68888738	G	A	0.437	genotype count	2473	3824	1498	*rs4148008*	Willer *et al*. [18]
						TC/HDL-c	4.17	4.215	4.282	(*r*^2^ = 0.98)	(24097068)
						triglyceride/HDL-c	0.0448	0.0460	0.0464		

Based on the results of the regression analyses of training sets (*n* = 6314) during the 10-fold cross-validation, we calculated the wGRS using the four SNPs and applied the wGRS to corresponding test sets (*n* = 702). The regression slopes of the six SNPs using the training sets were listed in electronic supplementary material, table S1 with their *p*-values. After performing 10-fold cross-validation, we observed the relationship between wGRS and the cholesterol ratios. Our results showed upward trends for wGRS with increases of the TC/HDL-c in both the training set (*R*^2^ = 0.8864, *p* < 0.0001) and test set (*R*^2^ = 0.8279, *p* < 0.0001) (electronic supplementary material, figure S1*a*). The TG/HDL-c ratios also showed similar results with an *R*^2^ value of 0.8033 for the training set and 0.8248 for test set (electronic supplementary material, figure S1*d*). In addition, we also found upward trends in all other subgroup analyses using male and female subjects (*R*^2^ > 0.5, *p* < 0.0001) (electronic supplementary material, figure S1*b,c,e* and *f*).

**Figure 2.**
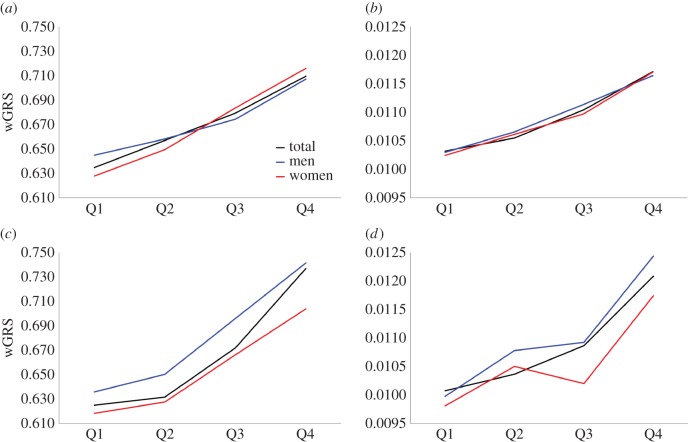
wGRS of TC/HDL-c and TG/HDL-c ratios in the SNP selection set and the final validation set. (*a*) wGRS with TC/HDL-c using the SNP selection set; (*b*) wGRS with TC/HDL-c using the final validation set; (*c*) wGRS with TG/HDL-c using the SNP selection set; (*d*) wGRS with TG/HDL-c using the final validation set.

Finally, regression slopes using the SNP selection set (*n* = 7016) were calculated to apply wGRS to the final validation set (*n* = 779) (*rs4420638*, 0.239 and 0.00249; *rs12421652*, 0.139 and 0.00233; *rs17411126*, 0.118 and 0.00227; *rs6589566*, 0.136 and 0.00274; *rs16940212*, 0.079 and 0.00095; *rs10852765*, 0.051 and 0.00066 for TC/HDL-c and TG/HDL-c ratio). As expected, wGRSs showed upward trends with increases of both the TC/HDL-c and TG/HDL-c ratios, similar to the results from the 10-fold cross-validation (figure 2). Although the wGRS of the third quantile for the female subjects was lower than that of the second quantile (figure 2*d*), the analyses for male and female subjects showed generally upward wGRS as the TC/HDL-c and TG/HDL-c ratios increased.

## 4. Discussion

To our knowledge, this is the first attempt made to build an SNP set for the prediction of both the TC/HDL-c and TG/HDL-c ratios in a Korean population using the wGRS method. The analysis scheme of the present study was designed based on previous studies [25,26]. Our results consistently showed an upward wGRS trend with increasing cholesterol ratios in all analyses, including final validation. These results indicated that the selected six SNPs (*rs4420638* (*APOC1*), *rs12421652* (*BUD13*)*, rs17411126* (*LPL*)*, rs6589566* (*ZPR1*)*, rs16940212* (*LOC101928635*) and *rs10852765* (*ABCA8*)) could be used for the prediction of both TC/HDL-c and TG/HDL-c ratios in a Korean population.

In the present study, we established variety sample sets using a total of 7795 subjects. To confirm cholesterol values of our sample sets, we consulted a previous large-scale report of cholesterol using Korean subjects [27]. According to the previous report, the TC level and HDL-c of Korean men was slightly lower than that of women. By contrast, TG was higher in women than men population. Similar differences also could be found in all of our sample sets, indicating that our sample sets were suitable for cholesterol prediction study for Korean population. According to the results (electronic supplementary material, figure S1), our SNP set for cholesterol ratios prediction showed good prediction ability in analyses using total subjects (*R*^2^ > 0.8). However, prediction ability for men and women subjects was slightly lower than total samples in both analyses for TC/HDL-c and TG/HDL-c (*R*^2^ = 0.5050 and 0.6763 for men; *R*^2^ = 0.6162 and 0.7700 for women). Further sex-specific analyses might be helpful for more precise cholesterol prediction.

Several previous studies have shown the importance of the six selected SNPs and genes for cholesterol metabolism and various diseases. The *rs4420638* which is located in the APOE-APOC1-APOC4-APOC2 cluster showed a protective effect on LDL-cholesterol levels [28]. The *rs4420638* was also responsible for risk of coronary heart disease of Asian population [29]. The association of *LPL* with lipid variables and coronary artery disease has been reported many times [30–32]. One recent study has demonstrated that the *rs17411126*, which is linked to *rs326* in *LPL* (*r*^2^ = 1.00), was implicated in the increase of HDL-c and *APOA1* after a high-carbohydrate and low-fat diet in males of the Han Chinese population [33]. In addition, several studies have suggested that the *rs6589566* could be a marker for the risk of coronary artery disease [34–36]. Moreover, the apolipoprotein A5 haplotypes, including *rs6589566*, were implicated in the elevation of the TG/HDL-c ratio and the risk for metabolic syndrome in a Korean population [37].

The exact roles of *rs12421652* (linked to *rs11216126* in *BUD13, r*^2^ = 1.00), *rs16940212* (*LOC101928635*) and *rs10852765* (linked to *rs4148008* in *ABCA8, r*^2^ = 0.98) in lipid metabolism are not fully understood yet. The strong association between *rs16940212* and blood cholesterol level (TG and HDL-c) was reported in the previous study using Korean population [38]. However, previous studies have found several pieces of evidence between the gene and lipid metabolism. *ABCA8* might function as a transporter of lipophilic substrates such as the bioactive lipid leukotriene C4 [39]. In addition, differential lipid response to statins was observed in a previous association study that used SNPs in the *BUD13*–*APOA5* gene region [40]. Further studies may be needed to understand the effects of SNPs on genes and lipid metabolism.

A recent study suggested a marker set for the prediction of cholesterol levels using various models, such as Ridge Regression, Lasso and Hyper-Lasso, with a Caucasian population [41]. Another study identified 19 of the most significant SNPs among the markers in 17 lipid-related genes in a Hispanic population [42]. Unfortunately, we failed to find our selected six SNPs in both of the previous studies. This inconsistency may be caused by the genetic background differences between Koreans and other populations, and indicates that our SNP set may not be suitable for the prediction of cholesterol ratios in other populations.

In summary, we composed an SNP set to predict cholesterol ratios using four markers. Using these markers, the wGRS showed increases of both the TC/HDL-c and TG/HDL-c ratios during the 10-fold cross-validation process. These results were also replicated in further analysis using the final validation set, as predicted. Although the exact role of the four SNPs in lipid metabolism was not fully elucidated, the SNPs explained the cholesterol ratio variation well for a Korean population. Our results might provide valuable information for the prevention of various diseases, including cardiovascular diseases.

## Supplementary Material

Supplementary Table 1. P-values and regression slope of 6 SNPs in training sets for 10-fold cross-validation

## Supplementary Material

Supplementary Figure 1. wGRS of Total cholesterol/HDL-c and Triglyceride/HDL-c ratios in Training and Test sets.

## Supplementary Material

Genotype data of the six SNPs

## Data accessibility

The data supporting the present study was available in the electronic supplementary material.
