# Supplementary material for: Prediction of cholesterol ratios within a Korean population
Source: R Soc Open Sci. 2018 Jan 17;5(1):171204. doi: 10.1098/rsos.171204 (PMC5792909; doi:10.1098/rsos.171204)
Supplement: Supplementary Table 1. P-values and regression slope of 6 SNPs in training sets for 10-fold cross-validation [file rsos171204supp1.docx]

Supplementary Table 1. *P*-values and regression slope of 6 SNPs in training sets for 10-fold cross-validation

| Total cholesterol/HDL-c ratio | | | | | | | | | | | | | |  | |  | |  | |  | |  |
| --- | --- | --- | --- | --- | --- | --- | --- | --- | --- | --- | --- | --- | --- | --- | --- | --- | --- | --- | --- | --- | --- | --- |
| Training set |  | *rs4420638* | |  | *rs12421652* | |  | *rs17411126* | |  | *rs6589566* | |  | | *rs16940212* | | | |  | | *rs10852765* | |
|  |  | *P-value* | *Regression slope* |  | *P-value* | *Regression slope* |  | *P-value* | *Regression slope* |  | *P-value* | *Regression slope* |  | | *P-value* | | *Regression slope* | |  | | *P-value* | *Regression slope* |
| Set 1 |  | 6.64E-13 | 0.2399 |  | 6.92E-08 | 0.1426 |  | 1.02E-06 | 0.1275 |  | 2.05E-07 | 0.1342 |  | | 0.001 | | 0.0728 | |  | | 0.008 | 0.0563 |
| Set 2 |  | 6.87E-13 | 0.2413 |  | 3.25E-08 | 0.1458 |  | 2.43E-06 | 0.1229 |  | 6.07E-08 | 0.1401 |  | | 0.002 | | 0.0705 | |  | | 0.005 | 0.0603 |
| Set 3 |  | 1.81E-11 | 0.2242 |  | 7.19E-08 | 0.1419 |  | 1.99E-06 | 0.1239 |  | 2.30E-07 | 0.1332 |  | | 0.0002 | | 0.0829 | |  | | 0.006 | 0.0586 |
| Set 4 |  | 6.87E-13 | 0.2413 |  | 3.25E-08 | 0.1458 |  | 2.43E-06 | 0.1229 |  | 6.07E-08 | 0.1401 |  | | 0.002 | | 0.0705 | |  | | 0.005 | 0.0603 |
| Set 5 |  | 2.70E-13 | 0.2439 |  | 8.21E-08 | 0.1415 |  | 4.76E-06 | 0.1196 |  | 6.83E-08 | 0.1395 |  | | 0.0006 | | 0.0765 | |  | | 0.007 | 0.0580 |
| Set 6 |  | 2.12E-12 | 0.2335 |  | 6.71E-08 | 0.1429 |  | 7.45E-06 | 0.1167 |  | 5.93E-08 | 0.1399 |  | | 0.0002 | | 0.0845 | |  | | 0.008 | 0.0568 |
| Set 7 |  | 1.09E-13 | 0.2486 |  | 1.66E-07 | 0.1382 |  | 1.04E-05 | 0.1150 |  | 4.20E-07 | 0.1310 |  | | 0.001 | | 0.0719 | |  | | 0.007 | 0.0571 |
| Set 8 |  | 6.41E-13 | 0.2403 |  | 2.49E-07 | 0.1367 |  | 2.94E-07 | 0.1334 |  | 4.98E-07 | 0.1295 |  | | 0.0007 | | 0.0752 | |  | | 0.01 | 0.0552 |
| Set 9 |  | 8.74E-13 | 0.2409 |  | 2.79E-07 | 0.1365 |  | 6.67E-06 | 0.1175 |  | 2.00E-07 | 0.1341 |  | | 0.0003 | | 0.0806 | |  | | 0.01 | 0.0554 |
| Set 10 |  | 5.85E-13 | 0.2400 |  | 7.23E-08 | 0.1435 |  | 5.16E-06 | 0.1186 |  | 8.42E-08 | 0.1382 |  | | 0.0002 | | 0.0845 | |  | | 0.006 | 0.0582 |
|  |  |  |  |  |  |  |  |  |  |  |  |  |  | |  | |  | |  | |  |  |
| Triglyceride/HDL-c ratio | | | | | | | | | | | | | |  | |  | |  | |  | |  |
| Training set |  | *rs4420638* | |  | *rs12421652* | |  | *rs17411126* | |  | *rs6589566* | |  | | *rs16940212* | | | |  | | *rs10852765* | |
|  |  | *P-value* | *Regression slope* |  | *P-value* | *Regression slope* |  | *P-value* | *Regression slope* |  | *P-value* | *Regression slope* |  | | *P-value* | | *Regression slope* | |  | | *P-value* | *Regression slope* |
| Set 1 |  | 1.65E-10 | 0.002526 |  | 2.09E-13 | 0.002295 |  | 3.48E+14 | 0.002334 |  | 4.40E+18 | 0.002644 |  | | 0.0002 | | 0.000973 | |  | | 0.006 | 0.000693 |
| Set 2 |  | 9.19E-10 | 0.002427 |  | 2.01E-14 | 0.002376 |  | 1.98E+12 | 0.002161 |  | 4.77E+19 | 0.002714 |  | | 0.001 | | 0.000857 | |  | | 0.005 | 0.000705 |
| Set 3 |  | 1.54E-09 | 0.002394 |  | 2.95E-15 | 0.002465 |  | 1.75E+14 | 0.002369 |  | 2.46E+19 | 0.002742 |  | | 0.0002 | | 0.000996 | |  | | 0.007 | 0.000686 |
| Set 4 |  | 9.19E-10 | 0.002427 |  | 2.01E-14 | 0.002376 |  | 1.98E+12 | 0.002161 |  | 4.77E+19 | 0.002714 |  | | 0.001 | | 0.000857 | |  | | 0.005 | 0.000705 |
| Set 5 |  | 1.21E-10 | 0.002548 |  | 7.55E-15 | 0.002427 |  | 3.59E+13 | 0.002249 |  | 1.71E+19 | 0.002760 |  | | 4.06E-05 | | 0.001089 | |  | | 0.003 | 0.000746 |
| Set 6 |  | 2.21E-09 | 0.002366 |  | 3.84E-14 | 0.002379 |  | 5.87E+13 | 0.002229 |  | 2.30E+19 | 0.002755 |  | | 3.35E-05 | | 0.001100 | |  | | 0.007 | 0.000682 |
| Set 7 |  | 1.16E-10 | 0.002558 |  | 6.34E-14 | 0.002344 |  | 8.01E+13 | 0.002211 |  | 3.86E+18 | 0.002658 |  | | 0.0005 | | 0.000926 | |  | | 0.008 | 0.000668 |
| Set 8 |  | 5.33E-11 | 0.002607 |  | 4.98E-12 | 0.002173 |  | 4.74E+15 | 0.002418 |  | 1.47E+17 | 0.002606 |  | | 0.0006 | | 0.000914 | |  | | 0.009 | 0.000660 |
| Set 9 |  | 2.49E-10 | 0.002523 |  | 2.15E-13 | 0.002304 |  | 2.63E+13 | 0.002252 |  | 1.19E+18 | 0.002682 |  | | 0.0002 | | 0.001002 | |  | | 0.005 | 0.000704 |
| Set 10 |  | 1.57E-10 | 0.002542 |  | 7.50E-13 | 0.002275 |  | 9.89E+14 | 0.002304 |  | 2.39E+18 | 0.002680 |  | | 0.0001 | | 0.001027 | |  | | 0.004 | 0.000733 |

HDL-c, High-Density Lipoprotein cholesterol;
