# Supplementary material for: Prediction of cholesterol ratios within a Korean population
Source: R Soc Open Sci. 2018 Jan 17;5(1):171204. doi: 10.1098/rsos.171204 (PMC5792909; doi:10.1098/rsos.171204)
Supplement: Supplementary Figure 1. wGRS of Total cholesterol/HDL-c and Triglyceride/HDL-c ratios in Training and Test sets. [file rsos171204supp2.docx]

Supplementary Figure 1.


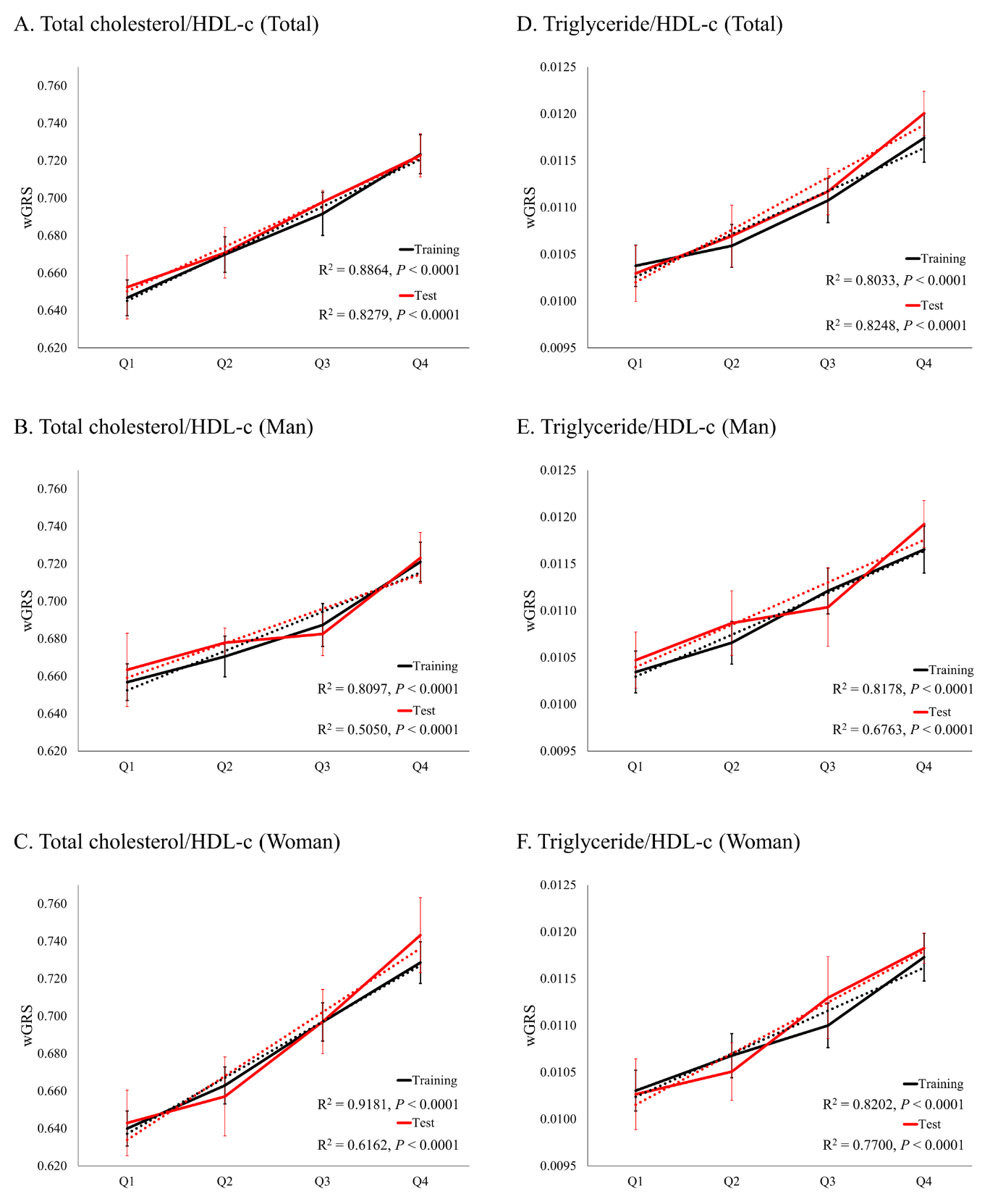


wGRS of Total cholesterol/HDL-c and Triglyceride/HDL-c ratios in Training and Test sets. Average wGRS was used for the graph. The standard deviation of wGRS in each quantile was used for error bars. Trend lines were shown as dotted lines with R^2^ and *P*-values.
